# Risk Factors for Human Salmonellosis Originating from Pigs, Cattle, Broiler Chickens and Egg Laying Hens: A Combined Case-Control and Source Attribution Analysis

**DOI:** 10.1371/journal.pone.0087933

**Published:** 2014-02-04

**Authors:** Lapo Mughini-Gras, Remko Enserink, Ingrid Friesema, Max Heck, Yvonne van Duynhoven, Wilfrid van Pelt

**Affiliations:** National Institute for Public Health and the Environment (RIVM), Centre for Infectious Disease Control (CIb), Bilthoven, the Netherlands; Cornell University, United States of America

## Abstract

Several case-control studies have investigated risk factors for human salmonellosis while others have used *Salmonella* subtyping to attribute human infections to different food and animal reservoirs. This study combined case-control and source attribution data into a single analysis to explore risk factors at the point of exposure for human salmonellosis originating from four putative food-producing animal reservoirs (pigs, cattle, broilers and layers/eggs) in the Netherlands. We confirmed that most human cases (∼90%) were attributable to layers/eggs and pigs. Layers/eggs and broilers were the most likely reservoirs of salmonellosis in adults, in urban areas, and in spring/summer, whereas pigs and cattle were the most likely reservoirs of salmonellosis in children, in rural areas, and in autumn/winter. Several reservoir-specific risk factors were identified. Not using a chopping board for raw meat only and consuming raw/undercooked meat were risk factors for infection with salmonellas originating from pigs, cattle and broilers. Consuming raw/undercooked eggs and by-products were risk factors for layer/egg-associated salmonellosis. Using antibiotics was a risk factor for pig- and cattle-associated salmonellosis and using proton-pump inhibitors for salmonellosis attributable to any reservoir. Pig- and cattle-associated infections were also linked to direct contact with animals and environmental exposure (e.g. playing in sandboxes). Eating fish, meat in pastry, and several non-meat foods (fruit, vegetables and pasteurized dairy products) were protective factors. Consuming pork and occupational exposure to animals and/or raw meats were protective against layer/egg-associated salmonellosis. We concluded that individuals acquiring salmonellosis from different reservoirs have different associated risk factors, suggesting that salmonellas may infect humans through various transmission pathways depending on their original reservoirs. The outcome of classical case-control studies can be enhanced by incorporating source attribution data and vice versa.

## Introduction

Salmonellosis is a major cause of human bacterial gastroenteritis and the second most reported zoonosis in the European Union (EU), after campylobacteriosis [1]. It has been estimated that more than 34,500 symptomatic infections with *Salmonella* spp. occur annually in the Netherlands (∼16.5 million population) [2], with Enteritidis and Typhimurium being the two most frequently isolated *Salmonella enterica* subsp. *enterica* serotypes, accounting for 29% (Enteritidis) and 47% (Typhimurium, including its monophasic variant 4,[5],12:i:-) of human cases in 2011 [3].

The overall number of culture-confirmed human *Salmonella* infections, particularly those caused by serotype Enteritidis, has decreased markedly over the last decades [3]. Even so, salmonellosis continues to contribute heavily to the disease burden of foodborne pathogens, both in terms of Disability Adjusted Life Years (DALYs) [2] and cost of illness [4], [5]. A particularly worrisome trend concerns the rapid emergence of *S*. 4,[5],12:i:-, which increased from 0.1% in 2004 to 20% in 2011 of all human *Salmonella* infections [3]. Human infections with the classical strains of *S*. Typhimurium have remained relatively stable over recent years [3], albeit with major outbreaks at both local [6] and national [7], [8] level. In addition, sudden explosions of serotypes of traditionally minor importance have been documented. An instance is the 2012 massive outbreak of *S*. Thompson linked to a rather unusual vehicle: smoked salmon [9]. This shows that the epidemiology of salmonellas is complex and dynamic; thus, a multi-tiered approach to control is needed, taking into account the different reservoirs, pathways and risk factors involved.

Consumption of raw/undercooked eggs has consistently been identified as the primary risk factor for human *S.* Enteritidis infection [10], [11]. This is in line with the relatively high prevalence of *S*. Enteritidis in layers [1], [3]. In contrast, transmission pathways for *S*. Typhimurium are less clear. A variety of risk factors has been identified, including consumption of beef [6], [12], pork [13], dairy products made with raw milk [7], exposure to animals [14] or to raw meats [10], and playing in sandboxes [10]. Yet, *S*. Typhimurium is mainly isolated in pigs and cattle [1], [3].

*Salmonella* source attribution is being performed in several countries to ascertain the main food-producing animal reservoirs towards which control efforts should be directed and to assess the impact of such interventions [15]–[20]. Classical case-control studies can only trace back the source of human infections up to the exposure (e.g. food consumption, contact with animals, etc.), which, however, may not point to the original reservoirs because of, for instance, cross-contamination. Combining source attribution and case-control data would allow us to reconstruct the underlying transmission pathway, from a given reservoir up to the point of exposure, providing more refined results than when performing separate analyses [21]–[23].

The objectives of this study were: 1) to attribute human salmonellosis cases to four putative food-producing animal reservoirs (pigs, cattle, broilers and layers/eggs); and 2) to combine the results of the attribution analysis with the available case-control data [10] to explore risk factors at the point of exposure for human salmonellosis caused by *Salmonella* subtypes attributable to pigs, cattle, broilers and layers/eggs.

## Materials and Methods

### Human data

Data of the so-called “CaSa” study, a case-control study on risk factors for human salmonellosis conducted in the Netherlands between April 2002 and April 2003 [10], formed the basis of the present study. In the CaSa study, a total of 1194 culture-confirmed cases of human salmonellosis (533 and 437 of which caused by *S.* Enteritidis and *S.* Typhimurium, respectively) were identified by the Dutch Regional Public Health Laboratories (RPHLs) through passive surveillance. Controls (*n* = 3165) were selected by frequency matching by age group (0–4, 5–17, 18–29, 30–44, 45–59, ≥60 years), sex, quarter of the year, and degree of urbanization (urban: >2500 addresses/km^2^; urbanized: 500–2500 addresses/km^2^ ; rural: <500 addresses/km^2^). For both cases and controls, information regarding food consumption, eating habits, kitchen hygiene, contact with animals, occupation, recreational activities, medication use, history of chronic diseases, and contact with people with gastroenteritis, was collected using a standardized questionnaire. Questions covered the seven days prior to symptom onset (cases) and questionnaire completion (controls). Missing values were handled using multiple imputation as described previously [10].

Case isolates were sent to the Dutch National Institute for Public Health and the Environment (RIVM), which serves as the EU and national reference laboratory for *Salmonella*, for serotyping and further phage typing of *S.* Enteritidis and *S.* Typhimurium isolates as described elsewhere [24]. Of the identified cases, 414 (168 and 197 of which caused by *S.* Enteritidis and *S.* Typhimurium, respectively) were enrolled in the study. Exclusion criteria were: 1) having travelled abroad with at least one overnight stay; 2) living outside the Netherlands; or 3) not returning the abovementioned questionnaire.

### Animal data

*Salmonella* isolates from four food-producing animal reservoirs, i.e. pigs (*n* = 1595 isolates), cattle (*n* = 734), broilers (*n* = 2930) and layers/eggs (*n* = 822) (available as Supporting Information, Table S1) were collected between 2001 and 2004 as part of diagnostic activities and a diversity of surveillance programmes on farms, slaughterhouses and at retail. These isolates were sent to the RIVM by the Regional Veterinary Services for serotyping and further phage typing of *S.* Enteritidis and *S.* Typhimurium isolates, as was done for the human isolates.

### Source attribution analysis

For the purposes of source attribution, all serotyped/phage typed *Salmonella* isolates from human cases that had occurred in the Netherlands between January 2002 and December 2003 (*n* = 3735 isolates), were used. Of these cases, 271 (7.3%) and 350 (9.4%) were discarded from the analysis because they were travel- or outbreak-related, respectively. Another 170 cases (4.6%) were discarded because their serotypes/phage types were only found in humans and not in any of the considered reservoirs. The modified Dutch model for source attribution [15] was then used to attribute the remaining 2944 sporadic and domestic cases to pigs, cattle, broilers and layers/eggs. These cases were infected with 164 different *Salmonella* subtypes that were found in at least one of the considered reservoirs. These subtypes included 94 serotypes along with 51 *S*. Typhimurium subtypes and 19 *S*. Enteritidis subtypes (Table S1).

The modified Dutch model has been presented in detail previously [15]. Briefly, the expected number of human infections caused by subtype *i* from reservoir *j*, denoted as *λ_ij_*, is given by
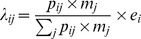



where *p_ij_* is the prevalence of subtype *i* from reservoir *j*, *e_i_* is the frequency of human salmonellosis cases of subtype *i*, and *m_j_* is the per capita annual food consumption (kg/person per year) for reservoir *j*. A sum over subtypes gives the total number of cases attributable to each reservoir. Details of model parameters are reported in Table 1.

**Table 1:** Parameters of the modified Dutch model for source attribution.

Parameter	Description/estimation	Source
*p_ij_*	prevalence of subtype *i* from reservoir *j,* given by *π_j_* • *r_ij_*	See below
*π_j_*	overall prevalence of *Salmonella* in reservoir *j,* given by Beta(*α*_j_+1,*β*_j_+1)	See below
*r_ij_*	relative frequency of serotype *i* in reservoir *j*, given by 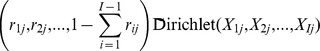 with *X_ij_* (*i* = 1, 2,…, *I*) being the isolates of subtypes *i* from reservoir *j*	Data
*α_j_*	*Salmonella*-positive samples from reservoir *j* in the Netherlands in 2002 (pigs 47; cattle 9; broilers 18; layers/eggs 18)	Bouwknegt *et al*. [49]
*β_j_*	total number of samples from reservoir *j* that have been tested for *Salmonella* minus *α_j_* in the Netherlands in 2002 (pigs 110; cattle 146; broilers 143; layers/eggs 116)	Bouwknegt *et al*. [49]
*e_i_*	frequency of human salmonellosis cases of subtype *i*	Data
*m_j_*	per capita annual food consumption (kg/person per year) for reservoir *j*, given by log(*m_j_*) ∼ Normal(*μ_j_*,1)	See below
*μ_j_*	average per capita annual food consumption for reservoir *j* in the Netherlands in 2002 (pig 42.2 kg; cattle 19.2 kg; broilers 17.3 kg; layers/eggs 13.9 kg)	Eurostat*; van Horne [50]

* Database "Food_ch_concap": Gross human apparent consumption of main food items per capita (http://epp.eurostat.ec.europa.eu/portal/page/portal/eurostat/home).

It has been pointed out that the amount of food consumed does not in itself account for the actual degree of exposure to a given source, as some foods are more likely to be eaten raw/undercooked than others [15]. An additional parameter (*c_j_*), expressing the probability for foods from reservoir *j* to be eaten raw/undercooked by the population, was therefore included in the model. Parameter *c_j_* was modelled as Beta(*α_j_*+1, *β_ j_*+1), where *α_j_* corresponded to the number of controls in the CaSa study reporting to have consumed raw/undercooked foods of pig (*n* = 278), cattle (*n* = 780), broiler (*n* = 244) and layer/egg (*n* = 1244) origin, and *β_j_* is the total number of controls enrolled (*n* = 3165) minus *α_j_*. The final form of the modified Dutch model used here was
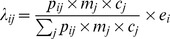



The model was implemented in @RISK (Palisade Corp., USA) by setting 100,000 iterations with the Latin hypercube sampling technique and a seed of 1. For every subtype, the model estimated a relative probability (*Pr*) to originate from each of the four reservoirs, as given by *λ_ij_*/∑*_j_λ_ij_*. Differences in *Pr* values for pigs, cattle, broilers and layers/eggs were tested between age groups, urbanization degrees, gender and seasons (spring/summer, i.e. April–September 2002 vs. autumn/winter, i.e. October 2002–March 2003) of the 414 salmonellosis cases enrolled in the CaSa study (Table S2) using Kruskal-Wallis or Mann-Whitney U tests, as appropriate (*α*-level: 0.05).

### Risk factor analysis

One-hundred and thirty-one putative risk factors were tested for association with the occurrence of salmonellosis of probable pig, cattle, broiler and layer/egg origin using *Pr*-weighted logistic regression models. A separate model for each reservoir was constructed. An equal probability weight of one was assigned to the controls, while the salmonellosis cases were weighted according to the reservoir *Pr* values for the subtype in question. Weights are often used in regression analysis to account for unequal probabilities of selection of sample units, as well as non-coverage, non-response and other unbalances of the sampled population that might lead to departures of the sample from the population [25]. Thus, by weighting the cases in our regression models according to their *Pr* values, the (sample) distribution of cases in the case-control study was adjusted to make it reflect the (population) distribution of cases that were more likely to originate from each of the reservoirs, as revealed by the source attribution analysis. As a result, factors associated with the occurrence of human salmonellosis in the *Pr*-weighted models could be interpreted as reservoir-specific risk factors.

Regression models were built in stepwise fashion. Variables showing a p-value ≤0.10 for the association with the outcome in the single-variable logistic regression analysis were selected for inclusion in a multivariable logistic regression model for each reservoir. Next, variables were dropped one by one if they showed a p-value ≥0.05 and their exclusion from the models did not influence the association of the other covariates. The population attributable risk (PAR) of each significant risk factor from the final model was then calculated based on adjusted odds ratios (ORs) and the prevalence of exposure in cases. Similarly, 95% confidence intervals (95%CI) of PARs were derived from the CIs of the adjusted ORs [10], [21]. All final regression models showed an overall statistical significance (likelihood ratio χ^2^ test, p<0.05) and goodness-of-fit (Hosmer-Lemeshow test, p>0.05). The level of education (categorized like in [10], [21]) was always included in the regression models to control for confounding in addition to the frequency-matched variables. Similar to previous analyses [10], [21]–[23], all possible two-way interactions between each of the significant risk factors of the reduced multivariable model and the age group, gender, season, and urbanization degree were tested for significance in a separate logistic regression model. This model included an interaction term at a time along with the corresponding original variables, while holding all other frequency-matched variables and the level of education constant. However, as no significant interaction was found, the reduced multivariable model was not expanded to include any of the tested interaction terms. In addition to the *Pr*-weighted models for each reservoir, an overall unweighted model was developed to identify risk factors for human salmonellosis as a whole. Finally, the multivariate shared relationships between the significant risk factors and the reservoir *Pr*s were explored altogether using canonical correlation analysis (CCA). Statistical analyses were performed using STATA 13 (StataCorp, USA).

## Results

### Attribution of human salmonellosis

The modified Dutch model estimated that the majority of the 2944 sporadic, domestic cases of salmonellosis that occurred in the Netherlands during 2002–2003, originated from layers/eggs (50.1%, 95%CI: 47.8–51.7%), followed by pigs (39.9%, 95%CI: 38.3–41.8%), cattle (6.2%, 95%CI: 5.5–7.7%) and broilers (3.8%, 95%CI: 2.9–4.6%). Cases infected with the two most prevalent serotypes, Enteritidis (*n* = 1361) and Typhimurium (*n* = 1009), were predominantly attributed to layers/eggs (89.5%, 95%CI: 85.1–92.9%) and pigs (81.8%, 95%CI: 78.5–84.3%), respectively. The remaining 574 cases infected with other serotypes were mainly attributed to pigs (49.24.8%, 95%CI: 46.45–51.99%) and layers/eggs (33.7%, 95%CI: 30.7–36.7%).

Median percent *Pr* values by age group, gender, season and urbanization degree are reported in Table 2. Significant age effects were found in *Pr* values for pigs (p = 0.003), cattle (p = 0.003), and layers/eggs (p = 0.001). The probability for pigs and cattle to act as reservoirs of salmonellosis was highest among children and adolescents, whereas that for layers/eggs was highest among adults. The probability to acquire infection from cattle was significantly higher for males than for females (p = 0.006). Significant seasonal effects were found in *Pr*s for pigs, cattle and layers/eggs. In autumn/winter vs. spring/summer, people had a significantly higher probability to acquire *Salmonella* infection from pigs (p<0.001) and cattle (p<0.001), whereas the inverse was found for layers/eggs (p<0.001). Significant effects of urbanization were found for all reservoirs. The probability for pigs and cattle to be reservoirs of salmonellosis was highest in rural areas (pigs, p<0.001; cattle, p = 0.001), whereas that for broilers and layers/eggs was highest in urban areas (broilers, p = 0.034; layers/eggs, p<0.001) (Table 2).

**Table 2:** Median percent probabilities for human salmonellosis cases to originate from pigs, cattle, broilers or layers/eggs according to age group, gender, urbanization degree, and season.

	Pigs	Cattle	Broilers	Layers/eggs
Age group (years)				
0–4	79%	5%	2%	7%
5–17	79%	7%	2%	12%
18–29	12%	3%	2%	76%
30–44	46%	3%	2%	45%
49–59	9%	3%	2%	83%
≥ 60	16%	3%	2%	29%
**Gender**				
Male	78%	5%	2%	12%
Female	30%	3%	2%	20%
**Urbanization degree**				
Urban (>2500 addresses/km^2^)	9%	2%	2%	86%
Urbanized (500–2500 addresses/km^2^)	72%	4%	2%	20%
Rural (<500 addresses/km^2^)	79%	6%	1%	6%
**Season**				
Spring/summer (April-September 2002)	14%	4%	2%	73%
Autumn/winter (October 2002-March 2003)	79%	7%	2%	5%

The Netherlands, 2002–2003.

### Reservoir-specific risk factors

Significant food-related risk factors for human salmonellosis as a whole were: not cleaning chopping board when using it for raw meat and other foods (PAR 11.6%), consuming raw/undercooked meat (7.1%), and consuming raw/undercooked eggs (3.6%) (Tables 3 and 4). Looking at the *Pr*-weighted models, not cleaning the chopping board remained a significant risk factor for salmonellosis attributable to pigs (PAR 14.4%) and cattle (17.2%), while changing kitchen rags less than once a week became a significant risk factor for broiler-associated salmonellosis (12.4%). Consuming raw/undercooked meat remained a risk factor for salmonellosis attributable to all three meat-producing animal reservoirs, i.e. pig (8.6%), cattle (9.4%) and broilers (8.7%), whilst consuming raw/undercooked eggs or products containing raw/undercooked eggs were risk factors for layer/egg-associated salmonellosis only (3.6% and 5.8%, respectively).

**Table 3:** Adjusted odds ratios and 95% confidence intervals of the significant risk factors for human salmonellosis attributable to specific animal reservoirs and overall.

Risk factor (% of imputed missing values)	Overall	Pigs	Cattle	Layers/eggs	Broilers
Eating raw/undercooked meat (3.9)	**2.1 (1.4**–**3.1)**	**2.8 (1.7**–**4.6)**	**3.4 (1.7**–**6.6)**		**2.9 (1.1**–**7.2)**
Eating chicken (0.9)				0.7 (0.5–1.0)	
Eating pork (0.8)				0.6 (0.5–0.9)	0.5 (0.3–0.8)
Eating meat in pastry (5.0)	0.7 (0.5–0.9)	0.6 (0.4–0.8)	0.5 (0.3–0.9)		
Eating raw/undercooked eggs (0.3)	**2.6 (1.5**–**5.5)**			**2.6 (1.2**–**5.8)**	
Eating products containing raw/undercooked eggs (1.0)				**1.8 (1.1**–**3.1)**	
Eating fish (2.9)	0.7 (0.5–1.0)	0.6 (0.4–0.9)	0.4 (0.3–0.7)		
Drinking pasteurized milk (1.6)			0.6 (0.4–0.9)		
Eating pasteurized dairy products other than milk and cheese (1.8)	0.6 (0.4–0.8)	0.5 (0.4–0.8)	0.5 (0.3–0.8)	0.5 (0.4–0.8)	
Eating raw vegetables (2.0)	0.7 (0.6–0.9)	0.6 (0.5–0.9)			
Eating cooked vegetables (3.2)	0.6 (0.5–0.8)	0.6 (0.4–1.0)		0.5 (0.3–0.7)	0.4 (0.3–0.7)
Eating salad (1.5)					0.6 (0.4–0.9)
Eating fruit (2.3)	0.7 (0.5–0.9)		0.6 (0.4–0.8)	0.5 (0.4–0.7)	0.5 (0.4–0.8)
Eating chocolate (2.1)	0.6 (0.5–0.8)	0.7 (0.5–1.0)		0.5 (0.4–0.7)	0.5 (0.4–0.8)
Eating nuts (3.1)	0.7 (0.5–0.9)	0.7 (0.5–1.0)	0.6 (0.4–0.9)		0.7 (0.4–1.0)
Not cleaning chopping board when using it for raw meat and other foods (1.7)	**1.4 (1.1**–**1.7)**	**1.5 (1.1**–**2.1)**	**1.7 (1.1**–**2.6)**		
Changing kitchen rags less than once a week (1.3)					**1.5 (1.0**–**2.3)**
Owning a puppy (0.0)		**2.5 (1.2**–**5.1)**			
Owning more than one dog, at least one puppy (1.5)				**1.7 (1.1**–**2.5)**	**1.8 (1.1**–**2.7)**
Occupation with animals and/or raw meat (1.0)			**6.7 (3.0**–**22.2)**	0.1 (0.0–0.3)	
Using antibiotics (0.0)	**1.9 (1.2**–**3.1)**	**2.5 (1.5**–**4.4)**	**2.8 (1.4**–**5.4)**		
Using proton-pump inhibitors (0.0)	**5.1 (3.1**–**8.2)**	**6.5 (3.6**–**11.6)**	**5.6 (2.9**–**10.5)**	**4.7 (2.7**–**8.3)**	**8.2 (3.6**–**18.4)**
Using H2-receptor antagonists (0.0)	**3.5 (1.4**–**8.6)**			**4.8 (1.9**–**12.2)**	**6.8 (2.6**–**17.9)**
Playing in a sandbox (1.1)		**1.8 (1.2**–**2.7)**	**2.2 (1.3**–**3.5)**		
Contacting people with gastroenteritis outside the household (7.9)	**1.8 (1.2**–**2.5)**	**2.3 (1.5**–**3.6)**	**2.2 (1.3**–**3.6)**		**2.0 (1.2**–**3.5)**
Contacting people with gastroenteritis within the household (1.3)				**1.9 (1.2**–**3.0)**	

Odds ratios presented are also adjusted for age, sex, degree of urbanization, season, and level of education. Risk factors are in bold, protective factors in normal font. Estimates are based on 414 cases (168 and 197 of which caused by *S.* Enteritidis and *S.* Typhimurium, respectively) and 3165 controls.

**Table 4:** Population attributable risk and 95% confidence intervals of the risk factors for human salmonellosis attributable to specific animal reservoirs and overall.

Risk factor (% of imputed missing values)	Overall	Pigs	Cattle	Layers/eggs	Broilers
Eating raw/undercooked meat (3.9)	7.1% (4.2–9.1%)	8.6% (5.6–10.5%)	9.4% (5.7–11.3%)		8.7% (1.6–11.5%)
Eating raw/undercooked eggs (0.3)	3.6% (1.9–4.7%)			3.6% (1.0–4.9%)	
Eating products containing raw/undercooked eggs (1.0)				5.8% (1.2–8.6%)	
Not cleaning chopping board when using it for raw meat and other foods (1.7)	11.6% (3.4–18.0%)	14.4% (4.2–21.9%)	17.2% (3.9–25.8%)		
Changing kitchen rags less than once a week (1.3)					12.4% (1.0–20.1%)
Owning a puppy (0.0)		2.0% (0.6–2.7%)			
Owning more than one dog, at least one puppy (1.5)				6.8% (2.0–10.1%)	7.4% (2.2–10.7%)
Occupation with animals and/or raw meat (1.0)			0.4% (0.3–0.5%)		
Using antibiotics (0.0)	3.7% (1.3–5.2%)	4.7% (2.5–6.0%)	5.0% (2.4–6.3%)		
Using proton-pump inhibitors (0.0)	7.9% (6.7–8.7%)	8.4% (7.2–9.1%)	8.1% (6.5–9.0%)	7.8% (6.2–8.7%)	8.7% (7.2–9.4%)
Using H2-receptor antagonists (0.0)	1.7% (0.7–2.1%)			1.9% (1.2–2.2%)	2.1% (1.5–2.3%)
Playing in a sandbox (1.1)		10.7% (3.5–15.4%)	13.2% (6.2–17.5%)		
Contacting people with gastroenteritis outside the household (7.9)	6.6% (2.8–9.3%)	8.8% (5.2–11.1%)	8.4% (3.9–11.1%)		7.8% (2.3–11.0%)
Contacting people with gastroenteritis within the household (1.3)				6.1% (2.3–8.6%)	

Population attributable risk is based on the multivariable odds ratios (see Table 3). Estimates are based on 414 cases (168 and 197 of which caused by *S.* Enteritidis and *S.* Typhimurium, respectively) and 3165 controls.

Among the significant non-food-related risk factors for salmonellosis as a whole, using proton-pump inhibitors (PPIs) showed the largest PAR (7.9%), followed by contacting people with gastroenteritis outside the household (6.6%), using antibiotics (3.7%), and using H2-receptor antagonists (1.7%) (Tables 3 and 4). In the *Pr*-weighted models, using PPIs remained a significant risk factor for salmonellosis attributable to any of the four reservoirs, with PARs ranging from 7.8% (layers/eggs) to 8.7% (broilers). Using H2-receptor antagonists remained significant for salmonellosis attributable to layers/eggs (1.9%) and broilers (2.1%), and using antibiotics remained significant for salmonellosis of pig (4.7%) and cattle (5.0%) origin. Contacting people with gastroenteritis outside the household remained a significant risk factor for salmonellosis attributable to meat-producing animals, with PARs ranging from 7.8% (broilers) to 8.8% (pigs), whereas contacting people with gastroenteritis within the household was associated with salmonellosis of layer/egg origin (6.1%) (Tables 3 and 4). Four additional risk factors became significant in the *Pr*-weighted models: owning a puppy for salmonellosis of pig origin (2.0%); owning >1 dog and at least one puppy for salmonellosis of layer/egg (6.8%) and broiler (7.4%) origin; playing in a sandbox for salmonellosis of pig (10.7%) and cattle (13.2%) origin; and occupational exposure to animals/raw meats, which was a significant risk factor for cattle-associated salmonellosis (0.4%), but a protective factor for layer/egg-associated salmonellosis.

Significant protective factors for salmonellosis as a whole were: consumption of pasteurized dairy products other than milk and cheese (e.g. yoghurt), chocolate, raw vegetables, fruit, meat in pastry, nuts, fish, and cooked vegetables (Tables 3 and 4). These protective factors remained significant in most of the *Pr*-weighted models. Of note, eating fish and eating meat in pastry remained significant for pig- and cattle-associated salmonellosis. Two additional protective factors, i.e. drinking pasteurized milk and eating salad, became significant in the models for cattle and broilers, respectively. Moreover, consuming chicken became protective for layer/egg-associated salmonellosis, while consuming pork became protective for both layer/egg- and broiler-associated salmonellosis.

### Canonical correlation analysis

**Figure 1:**
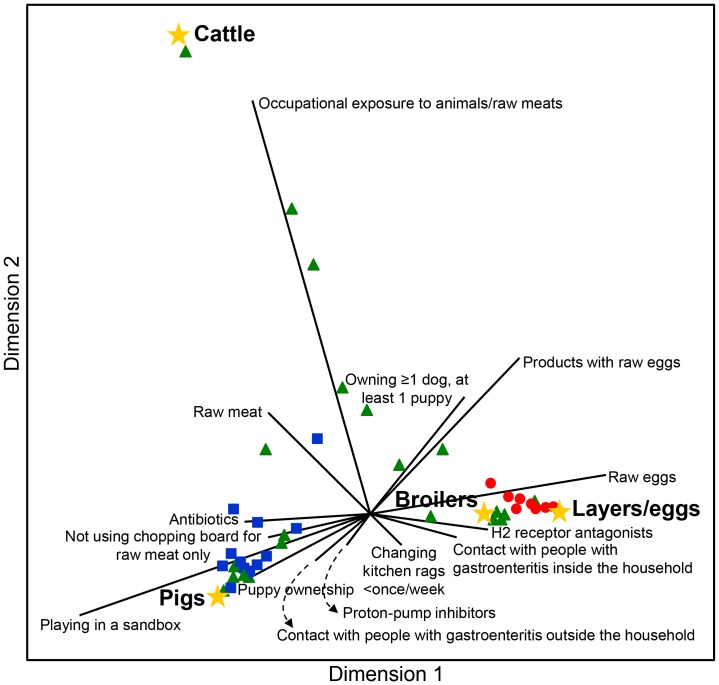
Plot of the first and second canonical dimension of the multivariate relationships between *Salmonella* subtype reservoir specificity (*Pr*s) and the significant risk factors. Blue squares: *S*. Typhimurium subtypes. Red dots: *S*. Enteritidis subtypes. Green triangles: other *Salmonella* subtypes. Yellow stars: reservoir centroids. Total variance explained by the first and second canonical dimensions is 82% and 15%, respectively.

The CCA defined three canonical dimensions which explained 82%, 15% and 3% of the total variance in the data, respectively. These canonical dimensions represent orthogonal, linear combinations of the original variables within each of the two sets (i.e. reservoir *Pr* values and risk factors) that best explain the variability both within and between these two sets of variables. In the scatterplot of the first two canonical dimensions (Figure 1), risk factors significantly associated with salmonellosis of pig, cattle, broiler and layer/egg origin are represented as arrows directed to where their correlations with the reservoir specificity are maximal. Like in a case-case comparison, risk factors in common to most reservoirs (e.g. PPIs) tend to disappear in the centre of the plot. In general, the CCA summarized and confirmed all the associations found in the logistic regression analysis. The point cloud of *S*. Enteritidis subtype *Pr*s was closest to layers/eggs and its density was relatively higher than that of *S*. Typhimurium, which was closest to pigs.

## Discussion

This is the first case-control study in which risk factors for human salmonellosis are investigated in relation to the attributable reservoirs using subtyping. Previous studies have mainly examined risk factors for salmonellosis according to different *Salmonella* subtypes [10]–[12], [14], [26], [27] and/or to specific populations at risk [12], [28]. Cummings *et al*. [29] have examined risk factors for human salmonellosis caused by bovine-associated subtypes in a similar way, although their attribution relied solely on the crude one-to-one matching (by geographical location, serotype and PFGE pattern) of human isolates with the contemporary bovine isolates, and a case-case approach was used. In the present study, quantitative risk modelling was used to attribute stochastically human cases to reservoirs, and the *Pr*-weighted case-control study design allowed for the identification of general risk factors for salmonellosis in addition to those factors that were reservoir-specific. These reservoir-specific risk factors provided an indication of the underlying transmission pathways by which salmonellas may reach humans from the different reservoirs.

### Reservoirs of human salmonellosis

Layers were estimated to be the most important reservoir of human salmonellosis in the Netherlands in 2002–2003, accounting for ∼50% of domestic and sporadic infections. This is in line with previous estimates based on the original Dutch model [3] and with other studies conducted in European countries using data from around that period [16], [30]. However, because of the significant drop in *S*. Enteritidis infections, the percentage of cases attributable to layers/eggs has decreased over the last decade in most European countries [15], [31], including the Netherlands, where layers/eggs in 2011 accounted for almost the same percentage of cases attributable to pigs (∼34%) [3]. Attribution analysis also confirmed that subtypes of *S*. Enteritidis are strongly associated with layers/eggs and those of *S*. Typhimurium with pigs. Yet, not all *S*. Typhimurium strains come from pigs and a considerable variation in host association within this serotype was found (Figure 1), a possible reflection of its ubiquitous nature.

The probability for human infections to originate from each of the reservoirs, as expressed by their *Pr* values, exhibited significant variation over age groups, gender, seasons and degrees of urbanization. For instance, adults were more likely to be infected by layer/egg-associated salmonellas, whereas pig- and cattle-associated salmonellosis was more likely to occur in the young. Moreover, cattle-associated salmonellosis was more likely to be acquired by males than by females. Although these differences are based on univariate analyses and should thus be interpreted with caution, they may reflect that men are generally more likely to be occupationally exposed to livestock and that different chances of exposure to foodborne and environmental sources exist at varying ages [32]. We also found that poultry-associated salmonellosis was more likely to occur in the warmest months of the year, whereas pig- and cattle-associated salmonellosis were more likely to occur in the coldest ones. The reasons of these seasonal differences are not entirely known. They may reflect a combination of factors, including the parallel *Salmonella* shedding trends in animal reservoirs [33], [34], a relatively larger effect of temperature on products with contaminated eggs combined with at-risk behaviours such as insufficient refrigeration or mishandling of foods [32], and the enhanced fitness to extreme environmental conditions of certain salmonellas such as *S*. Typhimurium, which is capable of rapidly recommencing normal growth after prolonged cold stress [35], [36].

Previous studies have found that chicken- and cattle-associated campylobacteriosis have differing urban-rural gradients, with chicken being the most likely reservoir for (foodborne) campylobacteriosis in people living in urban areas and cattle being the most likely reservoir for those living in rural areas, especially when considering children aged 0–4 years [21], [37], [38]. This was also reported for Shiga toxin-producing *Escherichia coli* (STEC) O157 infection [39], whereby suggesting a prominent role of environment-mediated transmission pathways for enteropathogens putatively shed by cattle in the countryside. We report here a similar effect of urbanization on salmonellosis, as pig- and cattle-associated salmonellas were more likely to infect people living in rural areas whereas poultry-associated salmonellosis was more likely to occur in urban areas.

### Reservoir-specific risk factors

The primary outcome of this study was to explore reservoir-specific, rather than subtype-specific, risk factors for salmonellosis. This was also pursued in the CCA, which not only confirmed the main findings of the regression analysis, but it also illustrated in a visually appealing way that risk factors are multidimensionally related to *Salmonella* subtype reservoir specificity. All risk factors were associated in an epidemiologically plausible way according to the reservoir in question. For instance, consumption of raw/undercooked meat (of any origin) was a risk factor for infections attributable to meat-producing animals (but not to layers/eggs), while consumption of raw/undercooked eggs and by-products was a risk factor for infections attributable to layers/eggs (but not to meat-producing animals). Moreover, consuming pork and chicken, together with occupational exposure to animals/raw meats, were protective against layer/egg-associated infections. Plausibly, a person may be somewhat more “protected” against infection from contaminated eggs when exposed to reservoirs other than layers, such as meat-producing animals. Furthermore, substandard kitchen hygiene and exposure to environmental sources such as playing in a sandbox were risk factors for salmonellosis attributable to meat-producing animals, particularly cattle and pigs. This agrees with our other finding that cattle- and pig-associated salmonellosis are more likely to occur in children and indicates that these salmonellas, the majority of which belongs to *S*. Typhimurium, are not only confined to foods, but may be widespread in the environment or be more capable of infecting people upon direct hand-mouth contact [10]. Indeed, occupational exposure to animals/raw meats was a risk factor for cattle-associated salmonellosis, supporting previous evidence [29] that contact with cattle increases the risk of bovine-associated salmonellosis and that more infections of cattle origin might result from direct contact than previously recognized.

Owning a puppy was a risk factor for pig-associated salmonellosis, but owning several dogs and at least one puppy was a risk factor for salmonellosis of poultry origin. The association between human salmonellosis and contact with dogs is already known [40], as dogs can carry (often asymptomatically) salmonellas, especially when kennelled or fed with raw foods [41]. Yet, the different associations found here are difficult to interpret; they may be either due to the different diets/feeding regimes of dogs or to at-risk behaviours undertaken by dog owners. For instance, feeding a household dog with (contaminated) dry food has been reported to be a risk factor for human *S.* Schwarzengrund infection [42], and attending veterinary facilities (as either an employee or a client) was linked to a multidrug-resistant *S*. Typhimurium outbreak in the United States, with splash exposure to dog faeces being the suspected means of transmission [43]. Further analyses considering the contribution of both food and non-food sources to human infections are needed in order to clarify the epidemiological role of pets in the directionality of transmission of salmonellas and other enteropathogens [22].

Using PPIs was a risk factor for salmonellosis of any origin, while using H2-receptor antagonists increased the risk of acquiring poultry-associated salmonellas only. Moreover, using antibiotics was a risk factor for salmonellosis of pig and cattle origins. While neutralization of gastric acidity by antisecretory drugs may enhance the survival of salmonellas during their passage through the stomach, antibiotics may alter the intestinal microflora in a way that favours infection [12], [21], [44]. However, it is also possible that receiving these medications is just a marker of people with gastrointestinal problems and increased susceptibility to infection in general. Besides, these people may be under more frequent medical attention and diagnostic thoroughness so that *Salmonella* infections are more likely to be detected. Nevertheless, differences in salmonellosis origin associated with different medications are difficult to discern. They might be due to the different therapeutic indications, side-effects and power/duration of effects of PPIs versus H2-receptor antagonists, as well as to the relatively stronger selection pressure by antibiotics towards salmonellas in meat-producing animals than in layers, for which antibiotic use in the EU is highly restrictive (Commission Regulation EC 1177/2006). This hypothesis is also supported by the much higher incidence of antibiotic resistance and emergence of multidrug-resistant strains in *S*. Typhimurium and *S*. 4,[5],12:i:- than in *S*. Enteritidis [45], [46].

Contact with people with gastroenteritis inside the household was a risk factor for layer/egg-associated infections, whereas contact outside the household was associated with infections attributable to meat-producing animals. Considering that contact with other gastroenteritis cases within the household may merely be a proxy for a common source of infection among the family members rather than real person-to-person transmission, this association further supports the notion of the more foodborne nature of *S*. Enteritidis and the ability of *S*. Typhimurium to spread through non-food pathways.

Consumption of fish, meat in pastry and several non-meat foods, including fruits, vegetables and dairy products (mainly yoghurt), were protective against infections attributed to different reservoirs. Possibly, people who had consumed fish or meat in pastry were less likely to eat other (potentially more contaminated) meat products. Although a diet rich in fruits, vegetables and dairy probiotics may have genuinely beneficial effects on general health by inhibiting bacterial growth, enhancing general immunity to infection, and altering the intestinal flora in a way that prevents infection, most controls returning the questionnaire may just be particularly motivated people with a generally healthier lifestyle.

The main limitation of the present study is related to the age of the data. From 2002–2003 to date, many changes have occurred in *Salmonella* epidemiology worldwide; thus, what was identified as risk factors here might not reflect entirely the actual situation anymore. Although our data are relatively outdated in order to identify potential targets for today’s *Salmonella* control activities, they represent a large, comprehensive set of information to develop our combined case-control and source attribution analysis in a robust way. The analytical approach we propose to study reservoir-specific risk factors for human salmonellosis will be of particular interest when more recent data will become available. This will allow the analysis to be updated as to corroborate and compare results. Moreover, the analytical approach presented here can, in principle, be extended to virtually all other zoonotic pathogens for which combining epidemiological and source attribution data can make a difference. This seems to be the case of *Campylobacter*, for which similar analyses have been performed using multilocus sequence typing [21]–[23]. However, the attempt to use *Pr* weights was unsuccessful with *Campylobacter* (personal communication of the authors). This may be due to the fact that associations of *Campylobacter* sequence types (STs) with specific reservoirs are much more dynamic and transient in time and space than those of *Salmonella* serotypes/phage types [47], which tend to adapt to specific reservoirs more consistently, showing a generally greater discriminatory power between sources than *Campylobacter* STs. For this reason it was only possible to run the analysis for *Campylobacter* using those cases infected with rarely occurring and highly reservoir-specific STs, whereas for *Salmonella* it was possible to use the whole (*Pr*-weighted) dataset.

As a last point, it has been postulated that repeated exposures to enteropathogens may lead to sufficient immunity to provide protection against (severe) clinical illness [10], [48]. In case-control studies, this "protective immunity" would lead to misclassification, as some controls could have been infected asymptomatically. As cases were identified by passive surveillance, they were likely to represent the most severe, symptomatic infections that occurred in the population. Thus, the risk factors identified here especially represent risk factors for severe salmonellosis.

## Conclusions

Several case-control studies have investigated risk factors for human salmonellosis caused by specific subtypes while other studies have used *Salmonella* subtyping to attribute human infections to different food and animal reservoirs. This study combined both these types of studies into a single analysis to explore risk factors at the point of exposure for human salmonellosis attributable to four putative food-producing animal reservoirs.

We confirmed that salmonellosis in the Netherlands in 2002–2003 was mainly attributable to layers/eggs, followed by pigs. However, we also found that poultry is the primary reservoir of salmonellosis for adults, for people living in urban areas, and during spring/summer time, whereas pigs and cattle are the primary reservoir for children, for people living in rural areas, and during autumn/winter.

Besides the identification of some universal risk factors for gastrointestinal infections in general (e.g. using PPIs), this study shows that risk factors for salmonellosis vary according to the attributable reservoir, suggesting that salmonellas may infect humans through various transmission pathways depending on their original reservoirs. This study also shows that the outcome of classical case-control studies can be enhanced by incorporating source attribution data, providing a valuable approach for supporting and generating hypotheses. Results also indicate that the general concept of *Salmonella* source attribution modelling makes sense epidemiologically and that reservoir-specific risk factors for salmonellosis are mirroring, to some extent, the underlying differences in the epidemiology of the two most important serotypes, *S*. Typhimurium and *S*. Enteritidis, albeit in a more refined and informed way.

## Supporting Information

Table S1 **Frequencies of** ***Salmonella*** **subtypes in humans (2002-2003) and animal reservoirs (2001-2004), The Netherlands.**
(XLS)

Table S2 ***Salmonella*** **subtypes from human cases included in the CaSa study with information on age, gender, urbanization degree and season.**
(XLS)
